# Ovaries and testes of *Lithobius forficatus* (Myriapoda, Chilopoda) react differently to the presence of cadmium in the environment

**DOI:** 10.1038/s41598-022-10664-4

**Published:** 2022-04-25

**Authors:** Izabela Poprawa, Łukasz Chajec, Alina Chachulska-Żymełka, Grażyna Wilczek, Sebastian Student, Małgorzata Leśniewska, Magdalena Rost-Roszkowska

**Affiliations:** 1 Faculty of Natural Sciences, Institute of Biology, Biotechnology and Environmental Protection, University of Silesia in Katowice, Bankowa 9, 40-007 Katowice, Poland; 2 Faculty of Automatic Control, Electronics and Computer Science, Silesian University of Technology, Akademicka 16, 44-100 Gliwice, Poland; 3 Department of General Zoology, Adam Mickiewicz University, Uniwersytetu Poznańskiego 6, 61-614 Poznan, Poland

**Keywords:** Cell biology, Ecology, Physiology, Structural biology, Zoology

## Abstract

Proper reproduction depends on properly functioning gonads (ovaries and testes). Many xenobiotics, including heavy metals, can cause changes in somatic and germ line cells, thus damaging the reproductive capacity. The aim of this study was to investigate the effect of the heavy metal cadmium on the gonads, including germ line and somatic cells. It is important to determine whether cell death processes are triggered in both types of cells in the gonads, and which gonads are more sensitive to the presence of cadmium in the environment. The research was conducted on the soil-dwelling arthropod *Lithobius forficatus* (Myriapoda, Chilopoda), which is common for European fauna. Animals were cultured in soil supplemented with Cd for different periods (short- and long-term treatment). Gonads were isolated and prepared for qualitative and quantitative analysis, which enabled us to describe all changes which appeared after both the short- and long-term cadmium treatment. The results of our study showed that cadmium affects the structure and ultrastructure of both gonads in soil-dwelling organisms including the activation of cell death processes. However, the male germ line cells are more sensitive to cadmium than female germ line cells. We also observed that germ line cells are protected by the somatic cells of both gonads.

## Introduction

Cadmium is a chemical element that occurs naturally in zinc and lead ores^1^. It is widely distributed in rocks (its average content is 0.03–0.22 ppm), from which it is released because of the weathering process and reaches the soil or water^2^. However, most of the cadmium in the environment is anthropogenic. Its sources are fossil fuels, industrial processes (especially iron, steel, and non-ferrous metals production), agriculture (fertilizers), transport, waste incineration and municipal wastewater^3–5^. There was no biological function of that chemical element in higher organisms^1^; however, cadmium-dependent carbonic anhydrase has been found in the marine diatom *Thalassiosira weissflogii*^6^. Like other heavy metals (e.g., zinc, mercury or lead), cadmium pollutes the environment and has a negative effect on living organisms due to its high toxicity^5,7,8^. Occurring in the natural environment, it can accumulate in various tissues and organs of plants and animals, passing through the successive links of the trophic chain^9^. Cadmium acts as a mitogen, promotes cancer in several tissues, stimulates cell proliferation, inhibits DNA repair, inhibits, or causes apoptosis (depending on concentration), causes autophagy and necrosis, and impairs reproduction^3,5,8,10,11^. Some organisms have detoxification mechanisms related to the accumulation of heavy metals in the cells of certain organs, e.g., midgut^12–15^. This group of animals includes myriapods taxa (Diplopoda, Chilopoda, Pauropoda, and Symphyla)^12–14,16^, animals recognized as one of the bioindicators in the analysis of environmental pollution^17^.

Reproduction is an essentially biological process leading to the formation of new organisms. This process is fundamental for an individual, but above all, for the survival of the species. The female and male gonads—the ovaries and the testes, respectively—are responsible for reproduction in gonochoric species such as centipedes (Chilopoda)^18,19^. Centipedes are still a poorly understood group of arthropods in terms of the structure and ultrastructure of the gonads. Occasional data indicate that their females have an unpaired tubular or sack-like ovary the anterior part of which is prolonged into a long, thin terminal filament. The posterior part of the gonad continues into a short oviduct that can divide into two branches^12,20,21^. Male centipedes have one pair of testes or one large, dorsally located testis that extends into a vas deferens^12,21^. The single, highly elongated male gonad that prolongs into the vas deferens, is present in *Lithobius forficatus*. In Craterostigmomorpha and Scolopendromorpha, the male gonad resolves into several small testicular follicles connected to the vas deferens^21^. Gametogenesis, the process responsible for the formation of reproductive cells, is very sensitive to the action of xenobiotics such as heavy metals or drugs. These substances may interfere with this process, leading to reduced fertility or infertility of animal and human organisms^8,22^.

Cadmium in soil influences the organisms that live in it at many levels of their body organization: from tissues, through the cells and organelles to enzymes and ATP/ADP levels. To better understand cadmium's effect on soil invertebrates, we chose the commonly distributed centipede *Lithobius forficatus* (Linneus, 1758), and we analyzed the changes that took place under the influence of short- and long-term exposure to this heavy metal contained in the soil. *L. forficatus* is a soil invertebrate common throughout Europe. It is easy to obtain and breed, and the well-known biology of this species allows for conducting not only histological but also ecotoxicological studies. By analyzing the changes in the organism caused by the presence of, for example, heavy metals in the soil, it is possible to assess how these xenobiotics will affect other soil animals, as well as animals of higher levels of the trophic chain. Our experiments extended the preliminary research conducted on *L. forficatus* by other scientists^23–29^. In our earlier papers, we described the changes under cadmium's influence in the midgut, the salivary glands, and the fat body of that species^30,31^. We also analyzed the effect of cadmium on mitochondria in both somatic and germ line cells^32^. As mentioned previously, it is necessary to analyze how cadmium affects the structure and ultrastructure of the gonads, including both germ line cells and somatic cells in ovaries and testes. Thus, the main aim of this study was to analyze and describe all alterations caused by short- and long-term cadmium intoxication in gonads (ovaries and testis) of *L. forficatus*. Emphasis was placed on the ultrastructural changes in the somatic and germ cells as well as on the activation of the cell death processes caused by this heavy metal. In addition, long-term exposure of centipedes to cadmium will help determine whether the changes that occur after short-term treatment will be more substantial or triggered by some regenerative mechanisms.

## Materials and methods

### Material

Adult specimens of *L. forficatus* (males and females) were collected in southern Poland, e.g., Żywiec (19°12′E, 49°42′N) and from parks near Poznań (16°55′E, 52°24′N) (no specific permission is required). Animals were cultured in 30 l aquaria (RT, humidity 60%, photoperiod 12:12) and acclimated to laboratory conditions for several weeks^29^. The specimens were fed ad libitum with *Chironomus* larvae bought from a fishing goods supplier. The soil used in laboratory culture and the experiment was commercial horticultural soil (Geolia, ref. no 45845884) with the chemical properties described in our previous paper^30^.

#### Experiment

**Figure 1:**
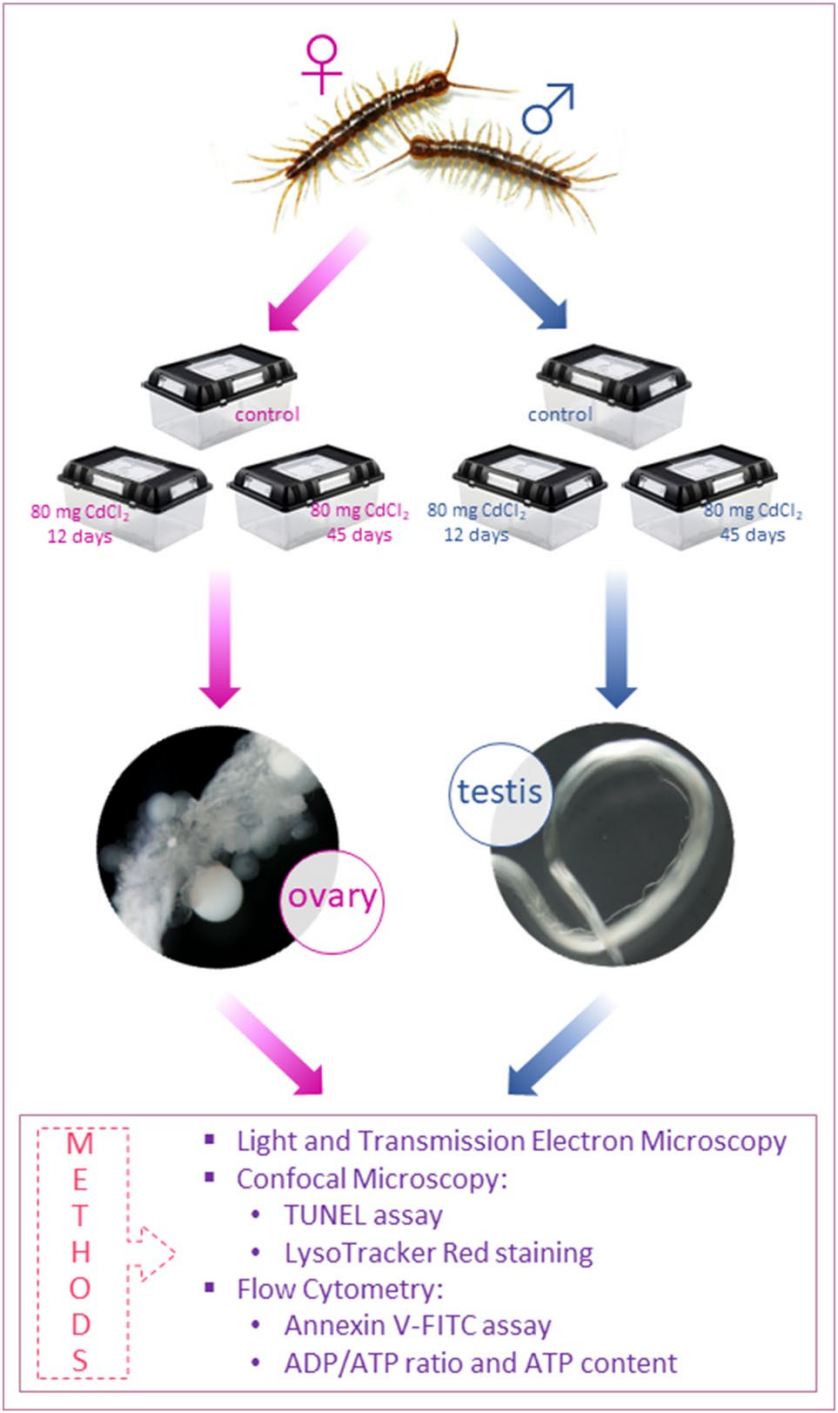
Scheme of experimental setup described in “Experiment”.

Animals were divided into three groups according to our previous research^30–32^: C—control group, animals bred in laboratory conditions as described above; 12Cd—animals bred in a horticultural soil containing 80 mg Cd kg^−1^ for 12 days (short-term cadmium exposure); 45Cd—animals bred in a horticultural soil containing 80 mg Cd kg^−1^ for 45 days (long-term cadmium exposure) (Fig. 1). Before sectioning, the animals were anesthetized, and ovaries and testes were dissected^30,31^. The cadmium concentration was selected based on an experiment conducted by Descamps et al.^28^, Vandelbucke et al.^29^, and our previous studies^30–32^. The number of specimens (females and males) used in the experiment is presented in Table 1.

**Table 1:** The number of adult specimens of *L. forficatus* dissected for each method. F—females, M—males.

Experimental group	Number of specimens examined				
	LM and TEM	TUNEL assay	LysoTracker Red	Flow cytometry Annexin V-FITC	ADP/ATP ratio
C, F/M	5/5	5/5	5/5	6/6	5/5
12Cd, F/M	5/5	5/5	5/5	6/6	5/5
45Cd, F/M	5/5	5/5	5/5	6/6	5/5

### Light and transmission electron microscopy

Testes and ovaries dissected from adult specimens were fixed with 2.5% glutaraldehyde and postfixed in 2% osmium tetroxide, dehydrated, embedded, and cut according to the standard method described by Rost-Roszkowska et al.^30,31^. Semi-thin sections after staining with 1% methylene blue in 0.5% borax were examined using an Olympus BX60 light microscope, while the ultra-thin sections after staining with uranyl acetate and lead citrate were analyzed using a Hitachi H500 transmission electron microscope at 75 kV.

### Confocal microscopy—qualitative analysis

#### Terminal deoxynucleotidyl transferase dUTP nick end labeling (TUNEL) assay

The TUNEL assay (In Situ Cell Death Detection Kit, TMR red; Roche, Basel, Switzerland) is commonly used to investigate the DNA fragmentation that occurs during apoptosis. The isolated gonads (testes and ovaries) were prepared according to manufacturer’s protocol and the method described in our previous work^30,33^. After staining the organs with a TUNEL reaction mixture, they were labeled with 1 mg/mL DAPI for the detection of nuclei (20 min, RT). Finally, the material was examined using an Olympus FluoView FV1000 confocal microscope with 40 × /NA 0.95 objective and using a 405 nm laser for the DAPI dye and 559 nm for the TMR red dye. Image sets were deconvolved in AutoQuant X3 (custom software developed by Bitplane Scientific Software, Zurich, Switzerland) using blind deconvolution. Three-dimensional data sets were examined as volume-rendered data sets using Imaris (Bitplane Scientific Software). Negative controls were conducted according to the producer’s labeling protocol.

#### LysoTracker Red staining: labeling autophagosomes and autolysosomes

LysoTracker Red staining selectively accumulates in strongly acidic organelles, so it is commonly used to investigate lysosomes and autolysosomes. The dissected testes and ovaries of adult specimens of *L. forficatus* from all experimental groups were incubated in 2.5 mmol/L LysoTracker Red DND-99 (Molecular Probes, L 7528; Thermo Fisher Scientific, Waltham, Massachusetts, USA) and 1 mg/ml DAPI (4′,6-diamidine-2′-phenylindole dihydrochloride) (Sigma-Aldrich) as was described by Rost-Roszkowska et al.^30^. The slides were observed using an Olympus FluoView FV1000 confocal microscope using a 559 nm laser for LysoTracker Red and 405 nm laser for the DAPI dye.

### Flow cytometry—quantitative analysis

#### Preparation of cell suspension

The dissected organs (testes and ovaries) isolated from specimens of each experimental group (Table 1) were crushed mechanically (Bead Bug microtube homogenizer) and suspended in 100 μL of PBS (pH 7.4). Then, the organs were homogenized and centrifuged as described in our previous paper^30^. The cell suspension was used for the flow cytometry according to the methods described below.

#### Viability assessment of gonad cells

Quantitative measures of viable, early, and late apoptotic and necrotic cells in ovaries and testis were obtained with the Annexin V-FITC (fluorescein isothiocyanate) Apoptosis Detection Kit (Abcam, № ab14085). This method is used to detect the early stages of apoptosis when translocation of phosphatidylserine (PS) groups from the inner to the outer leaflet of the plasma membrane occurs. Green fluorescence origins form cells bounded to the FITC-labeled Annexin V, while the red fluorescence origins form propidium iodide (PI). Thus, the distinction between necrotic cells (Annexin V-FITC–/PI +) and apoptotic cells (early apoptotic cells: Annexin V-FITC + /PI–; late apoptotic cells: Annexin V-FITC + /PI +) was enabled. Labeling was performed in dark according to the manufacturer’s protocol. The above-described cell suspension was analyzed in the Beckman Coulter Instrument FC 500 flow cytometer with a 488 nm argon laser. Fluorescence level results were examined with the CXP Analysis software for cytometric data.

### ATP level and ADP/ATP ratio—luminometry

The ApoSENSOR ATP Cell Viability Bioluminescence Assay Kit (BioVision, № K254 and ApoSENSOR ADP/ATP Ratio Bioluminescence Assay Kit (BioVision, № K255) were used to assess the status of both gonad cells. The analyses were conducted according to the manufacturer’s protocols. Determination of ATP concentration is based on the reaction of oxidative decarboxylation of luciferin catalyzed by luciferase, in the presence of high energy ATP and magnesium ions. The light intensity was measured at a wavelength of 562 nm. ADP level was measured by its conversion to ATP that is detected using the same reaction. Results were expressed as nmol ATP ∙ mg^−1^ protein. Protein content was measured according to Bradford^34^, using bovine albumin (protein content > 95%, Fluka) as standard.

### Statistical analyses

Statistical analyses were performed with STATISTICA 13 (StatSoft, Inc., 2016): normality (the Shapiro–Wilk test), homogeneity of variance (the Levene test), average and standard error (± SE). Eventually, the Tukey test (*p* < 0.05) was used to evaluate the significance of differences among experimental groups within gender.

## Results

Both the ovary and the testis in *Lithobius forficatus* are unpaired organs located on the dorsal side of the body. The ovary is prolonged anteriorly as a terminal filament, while its posterior end continues into a short oviduct. The testis is a very long tube-shape structure that forms two loops. Its anterior part extends into a terminal filament while the posterior part ends in a short vas deferens.

### Ultrastructural changes in ovaries of *L. forficatus* exposed to cadmium

#### Ovary in control animals

**Figure 2:**
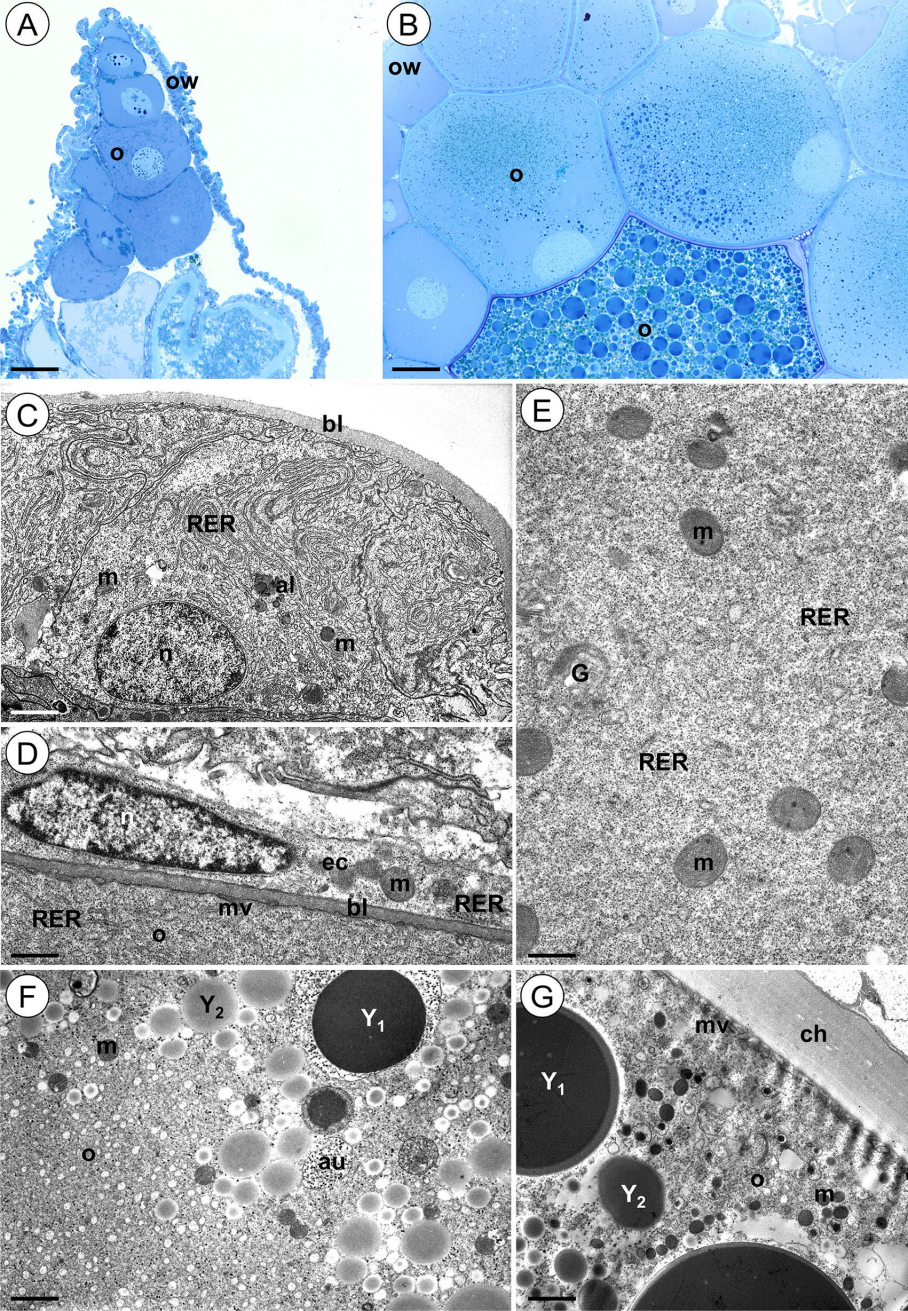
Ovary of *L. forficatus* from control group. (**A-B**) Longitudinal section through the ovary. Light microscope. (**C**) Cells of the ovary wall. TEM. (**D**) Epithelial cells surrounding oocyte. TEM. (**E**) Previtellogenic oocyte. TEM. (**F-G**) Vitellogenic oocyte. TEM. Autolysosome (al), autophagosome (au), basal lamina (bl), chorion (ch), Golgi complex (G), epithelial cells (ec), mitochondrium (m), microvilli (mv), nucleus (n), oocyte (o), ovary wall (ow), cisterns of rough endoplasmic reticulum (RER), yolk material (Y_1_, Y_2_). Scale bar: (**A**) 65 µm, (**B**) 81 µm, (**C**) 1.8 µm, (**D**) 1.2 µm, (**E**) 0.5 µm, (**F**) 1.5 µm, (**G**) 1.7 µm.

The ovary of the control animals is a sack-like structure filled with female germ cells accompanied by somatic cells (Fig. 2A, B). The ovary wall is composed of cubical or flattened somatic cells suspended by the basal lamina (Fig. 2C). The cytoplasm of these cells is rich in cisterns of the rough endoplasmic reticulum, mitochondria, and ribosomes (Fig. 2C). Sporadically autophagosomes and autolysosomes could be observed in somatic cells of the ovary wall (Fig. 2C). Each germ cell located in the ovary lumen is surrounded by a single layer of the flat somatic cells that rests on the basal lamina (Fig. 2D). The nuclei of these cells are flattened. Short cisterns of the rough endoplasmic reticulum, mitochondria, ribosomes, and Golgi complexes could be observed in their cytoplasm. The oolemma that surrounded the oocytes forms short microvilli (Fig. 2D). The cytoplasm of young oocytes (previtellogenic oocytes) is rich in ribosomes, mitochondria, short cisterns of the rough endoplasmic reticulum and Golgi complexes (Fig. 2E). In the cytoplasm of the vitellogenic oocytes the yolk material in the form of spheres of different electron density is accumulated (Fig. 2F,G). Sporadically the autophagosomes occurred in germ-line cells (Fig. 2F).

#### Ovary in animals after short-term Cd treatment (12Cd)

**Figure 3:**
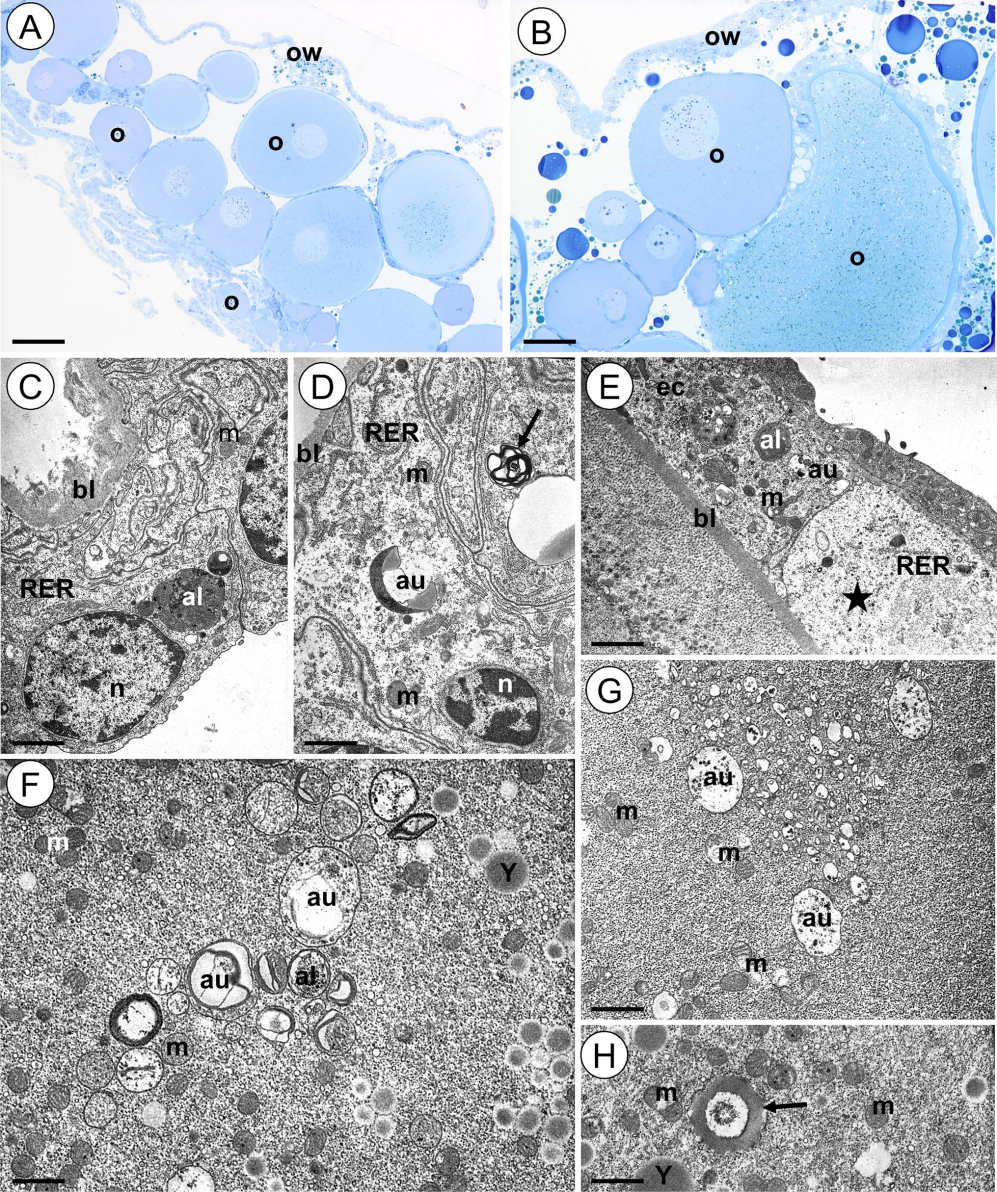
Ovary of *L. forficatus* treated with cadmium for 12 days (12Cd group). (**A-B**) Longitudinal section through the ovary. Light microscope. (**C-D**) Cells of the ovary wall. TEM. (**E**) Epithelial cells surrounding oocyte. TEM. (**F–H**) Oocytes. TEM. Autolysosome (al), autophagosome (au), basal lamina (bl), epithelial cells (ec), mitochondrium (m), nucleus (n), oocyte (o), ovary wall (ow), cisterns of rough endoplasmic reticulum (RER), yolk material (Y), necrotic cell (asterisk), spherite (arrow). Scale bar: (**A**) 90 µm, (**B**) 50 µm, (**C**) 2.3 µm, (**D**) 2.45 µm, (**E**) 2 µm, (**F**) 1.8 µm, (**G**) 1 µm, (**H**) 2 µm.

The ovary of the animals from the 12Cd group has the same shape as in the control group and retains its integrity (Fig. 3A, B). The entire cytoplasm of the majority of ovary wall cells is electron lucent with a small number of organelles (Fig. 3C, D). Cisterns of rough endoplasmic reticulum are short and their amount is lower than in the control group (Fig. 3C, D). Some mitochondria lose cristae and their matrix vacuolizes (Fig. 3D). Autophagosomes, autolysosomes and single spherites are visible in the cell cytoplasm in the ovary wall (Fig. 3C, D). Similar changes are observed in the somatic cells surrounding oocytes (Fig. 3E). Numerous autophagosomes, autolysosomes and degenerating mitochondria can be observed in these cells. Moreover, some of these cells show the necrosis. Their cytoplasm is electron lucent and the number of organelles is reduced (Fig. 3E). The oocytes show the intensification of autophagy; thus, numerous autophagic structures appear in their cytoplasm (Fig. 3F, G). Moreover, some mitochondria degenerate. They lose cristae and their matrix vacuolizes or becomes electron lucent (Fig. 3F, H). Single spherites appear in the cytoplasm (Fig. 3G). The yolk material is accumulated in the cytoplasm of vitellogenic oocytes. The yolk spheres are slightly smaller compared to those observed in oocytes in the control group.

#### Ovary in animals after long-term Cd treatment (45Cd)

**Figure 4:**
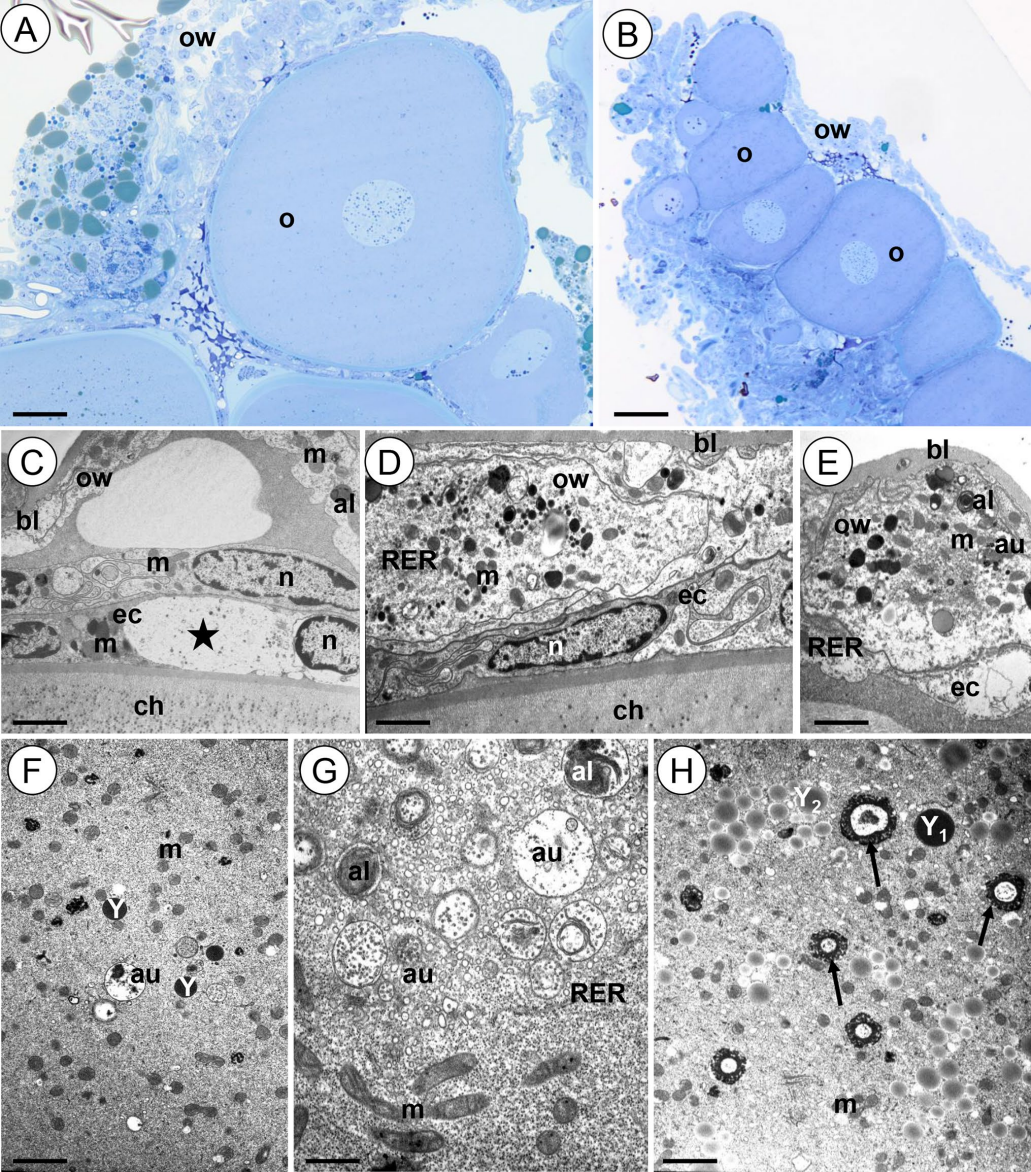
Ovary of *L. forficatus* treated with cadmium for 45 days (45Cd group). (**A-B**) Longitudinal section through the ovary. Light microscope. (**C-E**) Somatic cells of the ovary. TEM. (**F–H**) Oocytes. TEM. Autolysosome (al), autophagosome (au), basal lamina (bl), epithelial cells surrounding oocyte (ec), mitochondrium (m), nucleus (n), oocyte (o), ovary wall cells (ow), cisterns of rough endoplasmic reticulum (RER), yolk material (Y, Y_1_, Y_2_), necrotic cell (asterisk), spherite (arrow). Scale bar: (**A**) 40 µm, (**B**) 63 µm, (**C**) 1.6 µm, (**D**) 1.1 µm, (**E**) 1.45 µm. (**F**) 0.95 µm, (**G**) 1 µm, (**H**) 1 µm.

The ovary of the animals from the 45Cd group also retains its integrity and is filled with germ cells accompanied by somatic cells (Fig. 4A-B). The ovary wall cells look like those observed in the 12Cd group; however, only a few mitochondria show ultrastructural changes (Fig. 4C, E). Several of the somatic cells surrounding oocytes show signs of necrosis. They possess electron lucent cytoplasm and a reduced number of organelles (Fig. 4C, E). The second fraction of these cells has similar ultrastructure to that observed in the control group (Fig. 4C, D). Few autophagosomes and autolysosomes are observed in all types of somatic ovarian cells (Fig. 4C, E). Autophagosomes and autolysosomes observed in the cytoplasm of the oocytes are less numerous than in the 12Cd group (Fig. 4F, G), and moreover more intense autophagy occurs in previtellogenic oocytes (Fig. 4G). Only a few mitochondria show signs of degeneration, whereas most of them have the typical ultrastructure of the control group (Fig. 4F, G). Numerous spherites appear in the cytoplasm of vitellogenic oocytes (Fig. 4H). We did not observe a difference in the accumulation of yolk material between Cd12 and 45Cd groups.

### Ultrastructural changes in testis of *L. forficatus* exposed to cadmium

#### Testis in control animals

**Figure 5:**
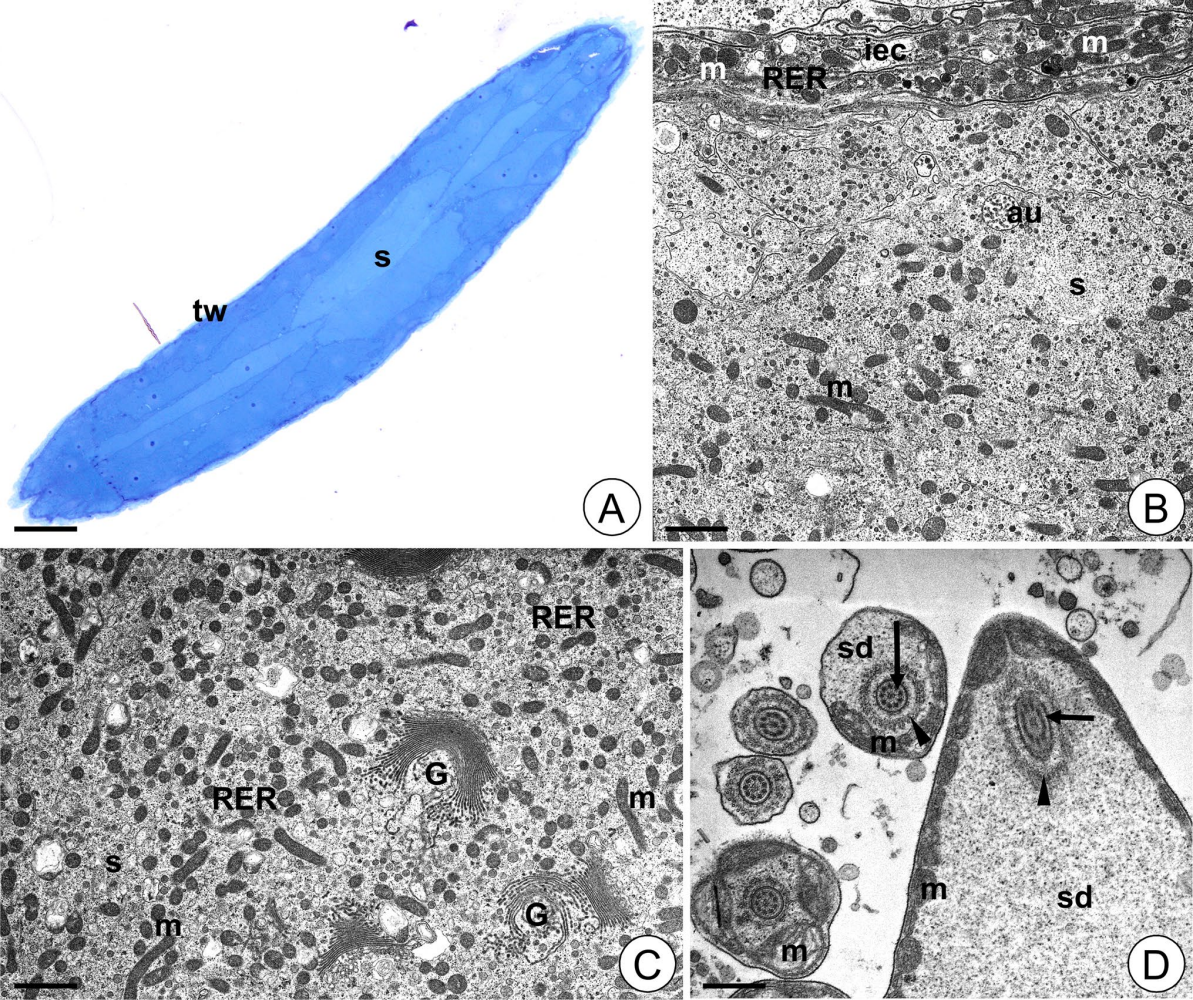
Testis of *L. forficatus* from control group. (**A**) Longitudinal section through the testis. Light microscope. (**B**) Testis wall. TEM. (**C**) Spermatocyte. TEM. (**D**) Spermatids. TEM. Autophagosome (au), Golgi complex (G), internal layer of epithelial cells (iec), mitochondrium (m), cisterns of rough endoplasmic reticulum (RER), spermatocyte (s), spermatid (sd), testis wall (tw), axoneme (arrow), microtubular sheet (arrowhead). Scale bar: (**A**) 120 µm, (**B**) 1.5 µm, (**C**) 1.1 µm, (**D**) 0.6 µm.

The testis of the control animals is a long, tube-like structure filled with male germ cells (Fig. 5A). The testis wall is composed of an internal layer of epithelial cells, circular muscles, thick connective tissue layers and an outer epithelial layer (Fig. 5B). The cytoplasm of epithelial cells is rich in ribosomes, mitochondria, and short cisterns of the rough endoplasmic reticulum (Fig. 5B). The spermatocytes have quite elongated egg-like shape (Fig. 5A). Their cytoplasm contains numerous mitochondria, ribosomes, short cisterns of the rough endoplasmic reticulum, and many highly curved Golgi complexes (Fig. 5B, C). In this stage mitochondria are distributed evenly throughout the cell. However, in elongated spermatids they are in the tail region close to the cell membrane forming the sheath of the axial filament (Fig. 5D). In the spermatid, microtubules form an axoneme that is surrounded by additional microtubules (microtubular sheet) that do not adhere to the axoneme (Fig. 5D).

#### Testis in animals after short-term Cd treatment (12Cd)

**Figure 6:**
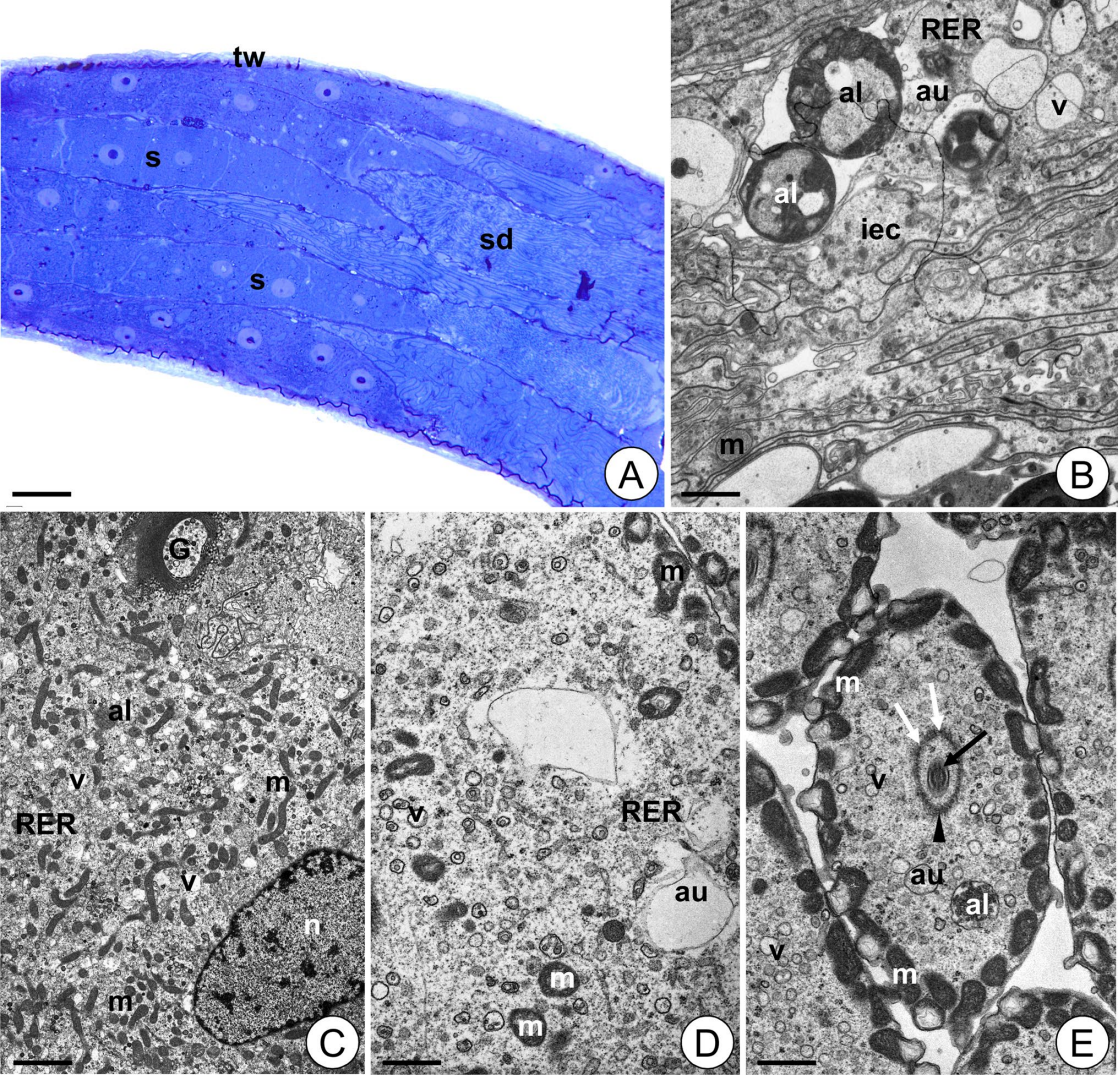
Testis of *L. forficatus* treated with cadmium for 12 days (12Cd group). (**A**) Longitudinal section through the testis. Light microscope. (**B**) Testis wall. TEM. (**C**) Spermatocyte. TEM. (**D-E**) Spermatids. TEM. Autolysosome (al), autophagosome (au), Golgi complex (G), internal layer of epithelial cells (iec), mitochondrium (m), nucleus (n), cisterns of rough endoplasmic reticulum (RER), spermatocyte (s), spermatid (sd), testis wall (tw), vacuole (v), axoneme (black arrow), microtubular sheet (arrowhead), gaps in microtubular sheet (white arrow). Scale bar: (**A**) 62 µm, (**B**) 1.25 µm, (**C**) 1.8 µm, (**D**) 0.7 µm, (**E**) Bar = 0.85 µm.

The testis of the animals from the 12Cd group retains its integrity and is filled with germ cells (Fig. 6A). Epithelial cells of the testis wall possess numerous vacuoles, autophagosomes and autolysosomes (Fig. 6B). Some mitochondria degenerate and lose cristae, and their matrix become electron lucent. Some autophagosomes, autolysosomes and vacuoles appear in the cytoplasm of spermatocytes. Sparse degenerating mitochondria can be observed in these cells (Fig. 6C). In the spermatids, numerous mitochondria show signs of degeneration. They partially lose their cristae, and vacuoles or lamellar bodies appear inside them (Fig. 6D, E). Some cisterns of the Golgi complexes inflate (Fig. 6C). Moreover, autophagosomes and autolysosomes are observed in the cytoplasm of spermatids (Fig. 6E). There are no disturbances in the structure of the axoneme but small gaps in the microtubular sheet are observed (Fig. 6E).

#### Testis in animals after long-term Cd treatment (45Cd)

**Figure 7:**
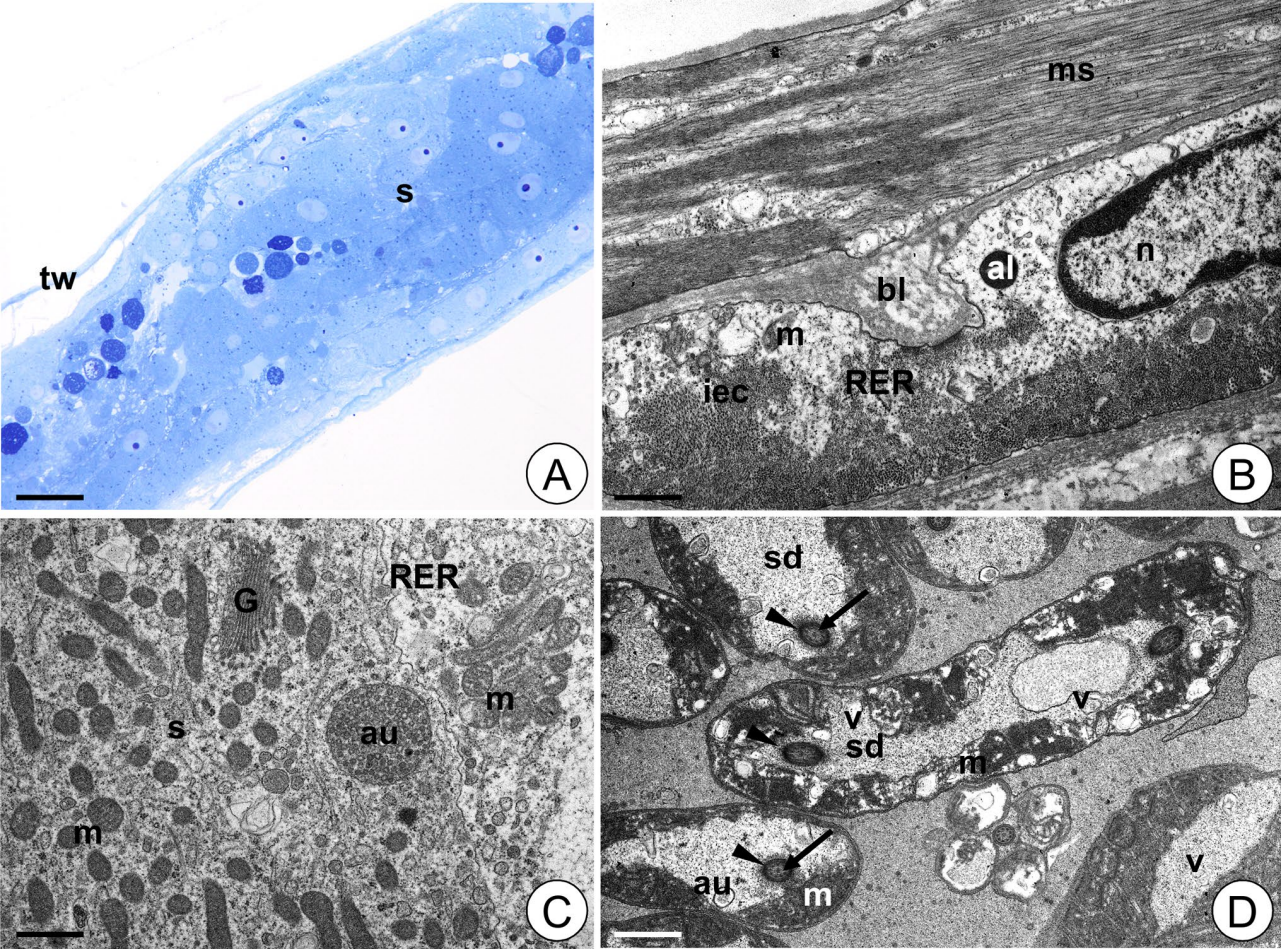
Testis of *L. forficatus* treated with cadmium for 45 days (45Cd group). (**A**) Longitudinal section through the testis. Light microscope. (**B**) Testis wall. TEM. (**C**) Spermatocyte. TEM. (**D**) Spermatids. TEM. Autolysosome (al), autophagosome (au), basal lamina (bl), Golgi complex (G),internal layer of epithelial cells (iec), mitochondrium (m), muscles (ms), nucleus (n), cisterns of rough endoplasmic reticulum (RER), spermatocyte (s), spermatid (sd), testis wall (tw), vacuoles (v), axoneme (arrow), damaged microtubular sheet (arrowhead). Scale bar: (**A**) 67 µm, (**B**) 0.95 µm, (**C**) 0.75 µm, (**D**) 0.75 µm.

The testis of the animals from the 45Cd group also retains its integrity and is filled with germ cells (Fig. 7A). Some of the testis wall epithelial cells show signs of necrosis. They possess electron lucent cytoplasm and a reduced number of organelles (Fig. 7B). Autophagosomes and autolysosomes appear in their cytoplasm (Fig. 7B). Ultrastructure of the spermatocytes is like that observed in the control group, but more autophagosomes, autolysosomes and few structurally altered mitochondria appear in the 45Cd group (Fig. 7C). Moreover, the Golgi complexes are less developed than in control and 12Cd groups and some of their cisterns inflate (Fig. 7C). In the cytoplasm of spermatids numerous degenerating mitochondria could be observed. They show similar changes to those observed in the mitochondria of the 12Cd spermatids (Fig. 7D). Single autophagosomes and vacuoles are also observed (Fig. 7D). No disturbances in the structure of the axoneme are observed while the microtubular sheet is damaged and its microtubules adhere to the axoneme (Fig. 7D).

### Autophagy in gonads of *L. forficatus* exposed to cadmium—qualitive analysis

**Figure 8:**
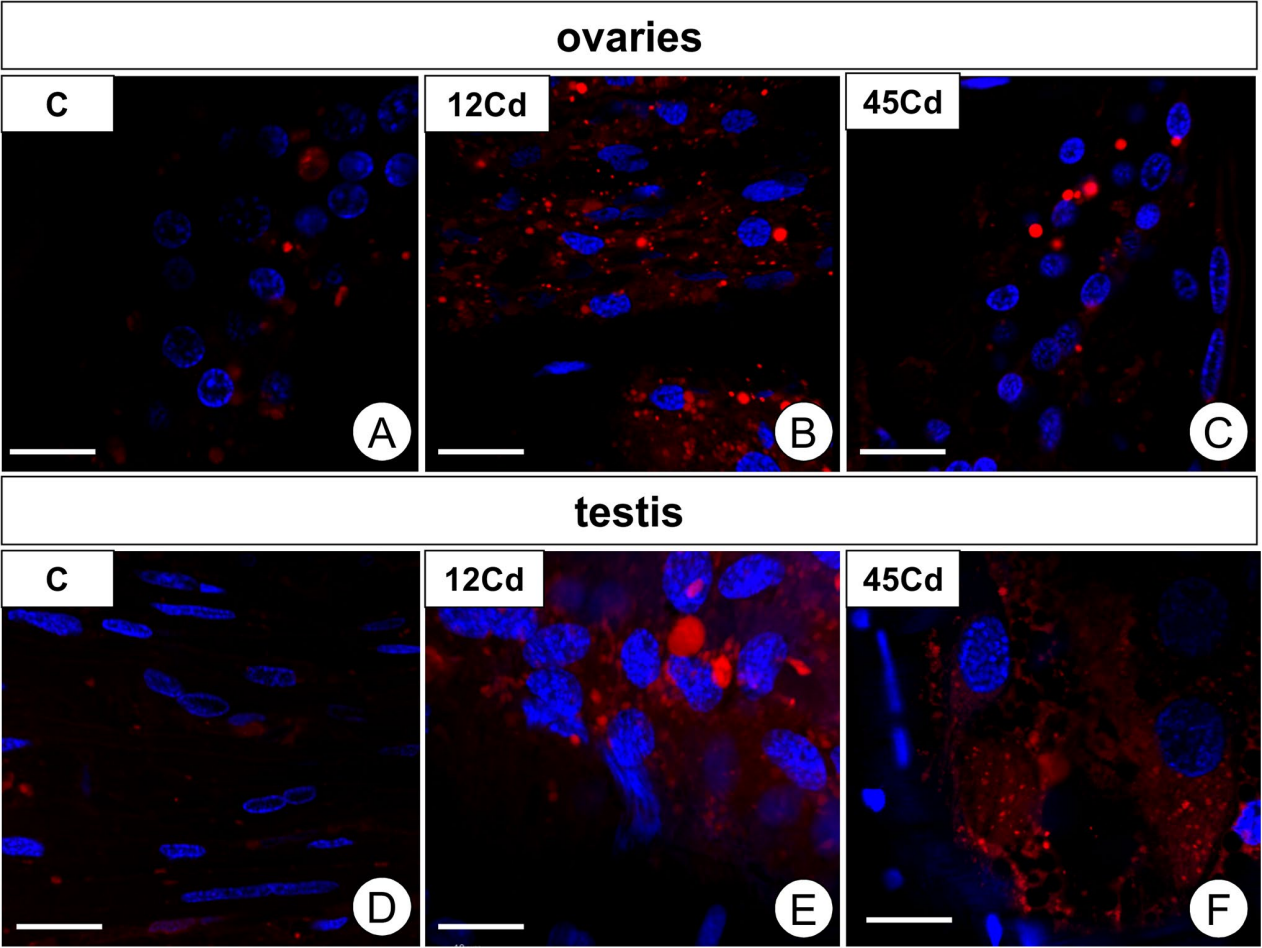
Autophagy in the ovary (**A-C**) and testis (**D-F**) of *L. forficatus* in control group (**C**), after 12 days of Cd treatment (12Cd) and after 45 days of Cd treatment (45Cd). 3D representation of accumulation of lysosomes and autolysosomes (red signals) in the ovary. Nuclei (blue signals). LysoTracker Red and DAPI staining, confocal microscopy. Scale bar: (A–D) 10 µm, (**E**, **F**) 5 µm.

Qualitative analysis using LysoTracker Red showed that the signals from acid organelles (autolysosomes, lysosomes) were stronger in both gonads in the 12Cd experimental group compared to the controls. After long-term cadmium treatment (45Cd), the emitted signals were slightly weaker than in Cd1 (Fig. 8A–F).

### Viability assessment of gonads in *L. forficatus* exposed to cadmium

**Figure 9:**
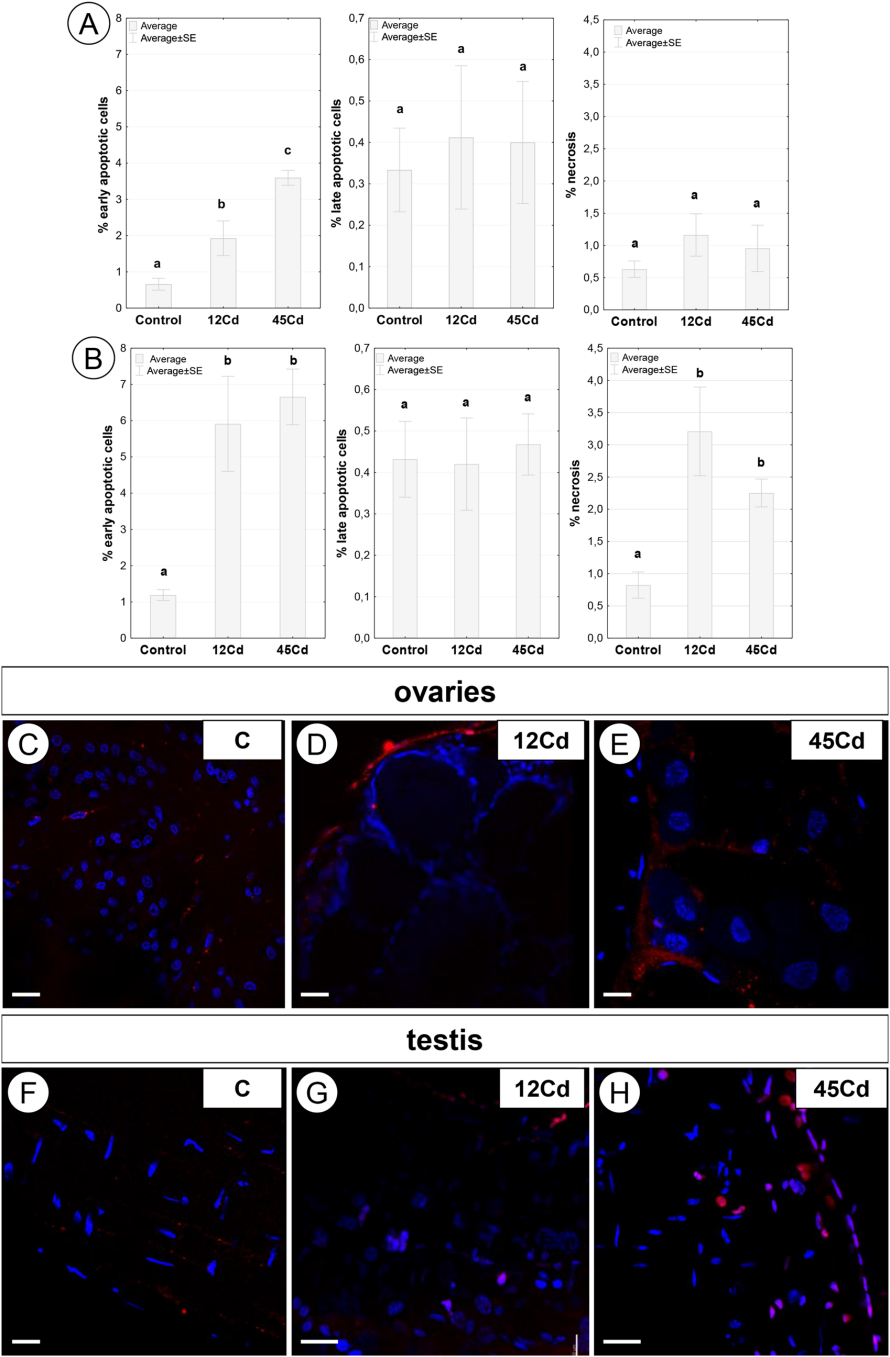
(**A**) Percentage of early apoptotic (Annexin^+^ PI^−^), late apoptotic (Annexin^+^ PI^+^) and necrotic (Annexin^−^ PI^+^) cells (x ± SE) in ovaries of *L. forficatus* from the control group and exposed to cadmium (12Cd, 45Cd). The different letters (a, b, c) indicate significant differences within each parameter (Tukey test, *p* < 0.05; n = 5–6). (**B**) Percentage of early apoptotic (Annexin^+^ PI^−^), late apoptotic (Annexin^+^ PI^+^) and necrotic (Annexin^−^ PI^+^) cells (x ± SE) in testis of *L. forficatus* from the control group and exposed to cadmium (12Cd, 45 Cd). The different letters (a, b, c) indicate significant differences within each parameter (Tukey test, *p* < 0.05; n = 5–6). (**C-E**) TUNEL assay and DAPI staining. Apoptosis in the ovary (**C-E**) and testis (**F–H**) of *L. forficatus* in control group (**C**), after 12 days of Cd treatment (12Cd) and after 45 days of Cd treatment (45Cd). Apoptotic nuclei (red signals), nuclei (blue signals). Scale bar: (**C-D**) 16 µm, (**E**) 10 µm, (**F**) 16 µm, (**G**) 14 µm, (**H**) 16 µm.

The quantitative analysis using flow cytometry revealed that more than a two-fold and nearly a three-fold increase in the number of early apoptotic cells was observed in the ovaries of individuals treated with cadmium for a period of 12 days (*p* = 0.035) and 45 days (*p* = 0.0003), respectively (Fig. 9A). The degree of severity of late apoptosis and necrosis in this organ of females was similar in all periods of cadmium treatment and like the control. Regardless of the time of exposure to cadmium in males there was observed an over six-fold increase in the percentage of early apoptotic cells (12Cd, *p* = 0.005; 45Cd, *p* = 0.004) and nearly threefold increase in the percentage of necrotic cells (12Cd, *p* = 0.001; 45Cd, *p* = 0.04) in testis, compared to the value of this parameter in individuals from the control group (Fig. 9B). The percentage of late apoptotic cells in gonads of males from 12 and 45Cd groups was similar as in the control group. Only after 12 days of exposure to cadmium were there statistically significant intergender differences in the percentage of early apoptotic cells (*p* = 0.002) and necrotic cells (*p* = 0.006) (Fig. 9A, B).

The qualitative analysis using confocal microscopy showed that the signals connected with the DNA fragmentation (the feature of apoptosis) in both gonads were stronger in specimens exposed to cadmium for 12 and 45 days compared to the control group. It enabled the somatic and germ cells to be distinguished. In animals from the control group, some of the apoptotic signals originated from the somatic and germ-line cells in testes, while in ovaries we managed to detect the apoptosis of the somatic cells (Fig. 9C, F). In animals from 12Cd experimental group we managed to detect the increase of signals emitted by apoptotic cells in both gonads (Fig. 9D, G). In animals exposed to cadmium for 45 days (45Cd), strong apoptotic signals were still emitted by both the somatic and germ-line cells in testes (Fig. 9H). The apoptosis concerned only somatic cells in ovaries of animals treated for 45 days with cadmium (Fig. 9E).

### ATP level and ADP to ATP ratio in gonads of *L. forficatus* exposed to cadmium

**Figure 10:**
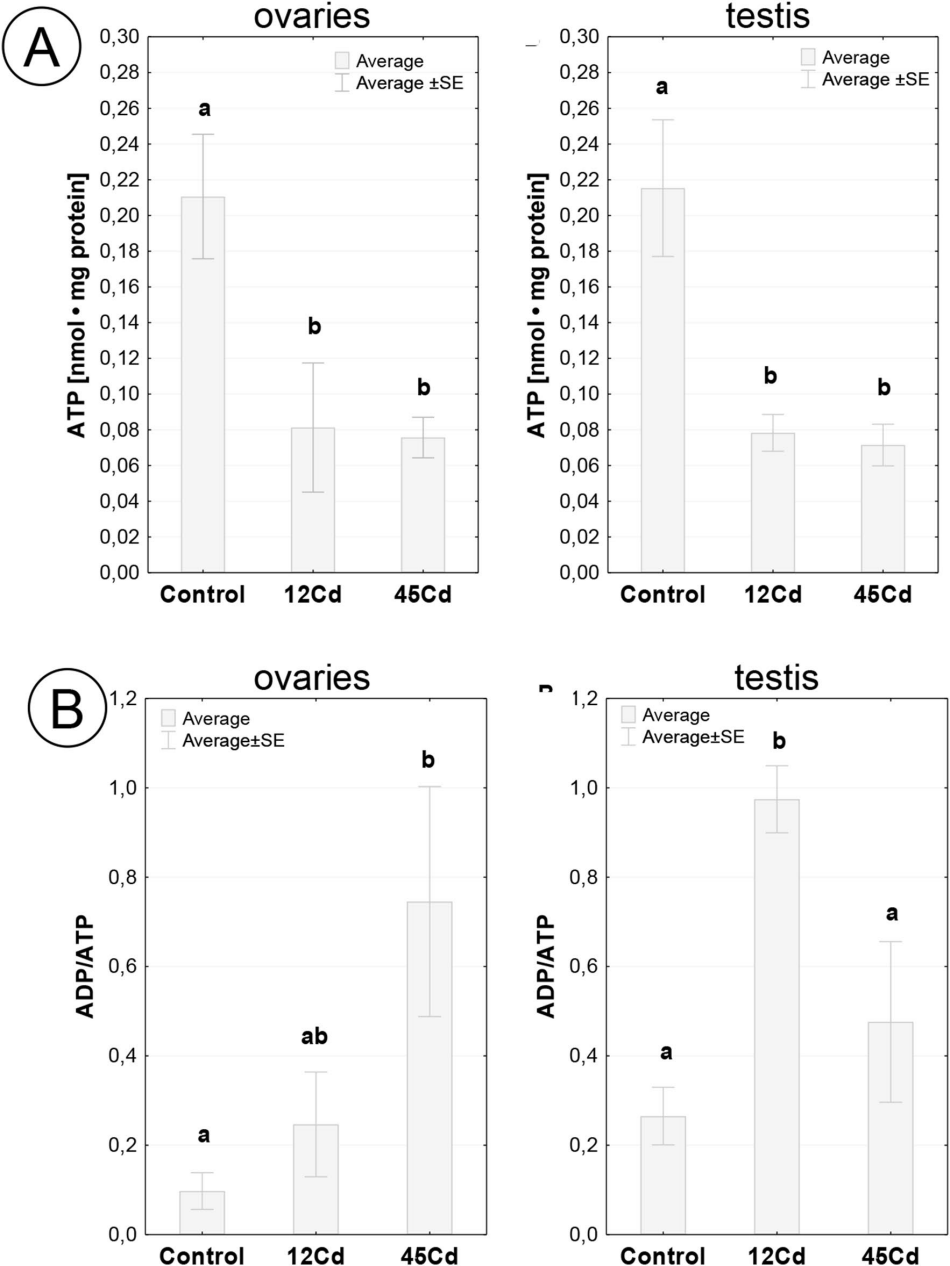
(**A**) Concentrations of ATP (x ± SE) in ovaries and testis of individuals from the control group and exposed to cadmium. The different letters (a, b) indicate significant differences among groups within each gonad (Tukey test, *p* < 0.05; n = 5–6). (**B**) ADP/ATP ratio (x ± SE) in ovaries (**A**) and testis (**B**) of individuals from the control group and exposed to cadmium. The different letters (a, b) indicate significant differences among groups within each gonad (Tukey test, *p* < 0.05; n = 5–6).

Regardless of gender and time of exposure to the metal, the ATP level in gonads of individuals intoxicated with cadmium was nearly three-fold lower compared to the control (12Cd, *p* = 0.039; 45Cd, *p* = 0.029) (Fig. 10A). There were no statistically significant differences in ATP concentration in ovaries and testes of individuals from complementary groups exposed to the metal. The ADP/ATP index in control group gonads was below 0.3. In females treated with cadmium for 45 days, the ADP/ATP ratio in ovary cells was more than seven-fold higher, compared to the control (*p* = 0.035), and over two-fold higher compared to the 12Cd group. In males the highest level of ADP/ATP index was in testes of individuals exposed to cadmium for 12 days (*p* = 0.018), whereas after 45 days of exposure, it decreased by almost two-fold, to a value not significantly different from the control (Fig. 10B). Only after 12 days of exposure to cadmium were there statistically significant intergender differences in ADP/ATP index (*p* = 0.015) (Fig. 10B).

## Discussion

Centipedes in their natural environment are exposed to various types of stressors, including heavy metals. One of them is cadmium, which is highly toxic to living organisms. It can enter the body directly from the environment through the body wall or food^35,36^. In our previous studies, we analyzed the effect of short- and long-term soil cadmium exposure on midgut, salivary glands, fat body, and mitochondria activity in somatic and germ cells in the centipede *Lithobius forficatus*^30–32^. Our research results indicated that different organs in the body might react differently to the same stressor at the same concentration and duration of exposure. Our latest research on the effect of cadmium in soil on *L. forficatus* gonads has shown that the ovaries and testes may react differently to the presence of this xenobiotic in the environment. Moreover, differences in the changes taking place were also observed between somatic and germ cells in male and female gonads.

The somatic cells that build the gonadal wall in both the ovary and the testis of the animals treated with cadmium for 12 and 45 days showed signs of degeneration. Their cytoplasm was electron lucent and poor in cell organelles. In addition, small vacuoles appeared in the cells of the testis wall. Similar, but on a smaller scale, changes were observed in the epithelial cells surrounding each oocyte. Such large degenerative changes in the cells of the gonad wall are related to the fact that these organs lie close to the body wall through which the xenobiotic enters the animal's body. Therefore, they are one of the first barriers to protect reproductive cells. Similar degenerative changes were observed in the somatic cells of the gonads of other invertebrates exposed to cadmium^37–39^. Histological studies showed that in *Blaps polycresta* Tschinkel 1975 (Coleoptera, Tenebrionidae), the follicular epithelium in the ovary was shrinking and ruptured under cadmium's influence. The testes of this species also showed a histopathological structure after the cadmium treatment, with shrinkage of acini and peritoneal membrane disintegration^39^. In cadmium-treated earthworm *Dendrobaena veneta* (Rosa, 1886), somatic gonadal cells underwent degenerative changes consisting of cell vacuolation, the appearance of irregular intercellular spaces, the disintegration of intercellular connections, and disintegration of cell membranes. These changes intensified both with the increase in cadmium concentration in the soil and the prolongation of this xenobiotic exposure^38^. Somatic cells in the ovaries and testes of animals are responsible for many functions related to the normal course of gametogenesis, including the synthesis of regulatory and storage substances, and in particular for the protection of germline cells^18,19^. The results of our studies on soil invertebrates confirmed such a statement.

Although protected by somatic gonadal cells, germ line cells are also exposed to the toxic effects of cadmium. The main organelles affected by this xenobiotic are the mitochondria. Both in oocytes and *L. forficatus* spermatocytes, changes in ultrastructure were observed only in a few mitochondria. In contrast, significant changes in their activity were observed, as described in detail in the previous paper^32^. Much greater ultrastructural changes were observed in the mitochondria of the spermatids of the studied species after both 12 and 45 days of exposure to cadmium. They partially lost their cristae, and vacuoles or lamellar bodies appeared inside them. Distension, vacuolation, and reduction of mitochondrial cristae are common changes in these organelles under the influence of cadmium. They were described in female and male germ cells of *D. veneta*^38,40–42^, in oocytes of *Palaemon serratus* (Pennant, 1777)^43^, in spermatids and spermatozoids of sea urchins and mussels^44–46^ as well as the somatic cells of different organs^30–32,37–39^.

Organelles of *L. forficatus* such as the rough endoplasmic reticulum and Golgi complexes showed signs of increased activity in oocytes of animals exposed to short- and long-term exposure to cadmium and in spermatocytes of animals treated with cadmium for 12 days. Degeneration of Golgi complexes occurred in spermatocytes after long-term xenobiotic exposure. On the one hand, the activity of these organelles in oocytes may be related to the process of yolk material accumulation (vitellogenesis)^47^. It may also be associated with the synthesis of metallothioneins and other Cd-binding proteins, which are cellular defense tools against cadmium^48–50^.

Single spherites were observed in the cytoplasm of oocytes and *L. forficatus* ovarian wall cells in the 12Cd group. Their number increases significantly in oocytes from the 45Cd group. Spherites are spherical granules in the form of concentric lamination that have been described in cells of the different invertebrate organs^14,15,17,51–55^. These structures can accumulate non-toxic and toxic substances such as calcium, magnesium, heavy metals, and organic material ^14,15,17,51,52^. In *L. forficatus*, spherites were observed in midgut cells in the control group and the experimental groups treated with cadmium^30^. These structures were not observed in the cells of the salivary glands, fat body^31^, or testes (present study). Considering that the spherites do not appear in the control group's ovarian cells, they appear in the 12Cd group, and their number increases significantly in the oocytes from the 45Cd group, we can assume that these structures accumulate cadmium in the studied species. Probably the accumulation of cadmium in spherites is one of the mechanisms that protect oocytes against the toxic activity of this heavy metal. Spherites are concentrically layered structures described in many organs of invertebrates through which the detoxification takes place. They contain both organic and non-organic compounds, including heavy metals. Thus, they are regarded as a barrier that inhibits the harmful influence of metal ions from reaching the entire organism^56^. They have also been described in soil myriapods belonging to centipedes and millipedes^13,15,27,57–59^. Spherites that accumulate in the oocytes of *L. forficatus* ovaries resemble that of class B containing cadmium, copper, and/or mercury^13^. Thus, we can conclude that the formation of spherites in oocytes of *L. forficatus* could be a protective mechanism against the effect of cadmium. Because of the fact that these structures were formed only in ovaries, these gonads are more protected than testes.

Literature data show that cadmium affects the process of vitellogenesis, i.e., the accumulation of yolk material in oocytes. The yolk is synthesized in smaller amounts, or its synthesis is inhibited^60,61^. Cadmium inhibits vitellogenesis, probably through a reduction in vitellogenin polypeptide synthesis^61^. *L. forficatus* individuals treated with cadmium synthesized yolk material, which accumulated in the oocytes. However, the yolk spheres were smaller than those observed in the oocytes of the control animals. These results may suggest that cadmium limits the synthesis of vitellogenins, which will hurt the studied species’ reproduction. Further studies, however, must be done to show how the mechanisms of vitellogenins synthesis are affected by exposure to cadmium.

During analysis changes in the microtubular sheet surrounding an axoneme in the spermatids of the cadmium-treated animals were observed. In the group treated with xenobiotics for 12 days, there were small gaps in the microtubular sheet, while 45-day exposure to cadmium destroyed this structure. The remaining microtubules adhered to the axoneme. Similar changes were noted in the microtubular cuff surrounding the nucleus of cadmium-treated earthworm *D. veneta* spermatids. In this case, these changes depended mainly on the time of exposure to a xenobiotic, and to a lesser extent on its concentration^42^. Changes within the microtubular sheet could be related to disturbances in microtubule polymerization leading to the formation of a damaged microtubular sheet or its absence^62^ or were related to depolymerization and destruction of microtubules in the already existing structure^63^.

Cell death can occur via several processes, such as apoptosis, autophagy and/or necrosis. Apoptosis is treated as an irreversible process connected with caspase activation and leading to dead cell removal without inflammation^64^. It is a common process responsible for the proper course of different developmental processes including germ-line cell functioning^65,66^. The apoptosis of male germ-line cells in mammalians has been reported to be involved in different steps of testicular development. The number of cells in their tubules is determined by a distinct balance between cell proliferation and apoptotic cell death. Thus, in vertebrates cells with genetic defects could be eliminated from the organ^67,68^. In the case of vertebrate and invertebrate ovaries, apoptosis has been described in ovarian follicles through embryonic and adult life. While during the vertebrate fetal life, this process concerns the oocytes, it has been detected in granulosa cells of secondary and antral follicles in adults^69,70^. Apoptosis of germline-derived cells is a common phenomenon observed in invertebrate ovaries and is necessary for the proper development of the oocyte^64,71–77^. Its role in female gonads is the removal of cells that are unable to differentiate into fertile eggs^64,77^ or to supply nutrients to the oocyte^77,78^. However, oocyte apoptosis is involved in the depletion of germ cells from the ovary and has a distinctly negative impact on mammalian female fertility^79,80^. In *L. forficatus* gonads, apoptosis was commonly observed in somatic cells, and it increased according to the increasing duration of animals’ exposure to cadmium. Apoptosis of germ-line cells occurred only in testes, while we did not manage to detect the apoptosis of oocytes. This suggests that somatic cells play a protective role in both gonads for the germ line cells, enabling them to survive. However, the apoptosis of male germ cells could relate to the fact that the majority of all male germ cells produced are discarded through the cell death process^66,68^. Cells that are damaged or badly developed are removed in this process^68,81^. The described degeneration of organelles in the cytoplasm of cells in both gonads of *L. forficatus* correlates with the previously described degenerative changes in mitochondria. We observed a decrease in the number of active mitochondria and an increase in non-active mitochondria^32^. Changes in the transmembrane mitochondrial potential together with ultrastructural changes of these organelles are alterations that lead to cell death^82–85^. Additionally, the mitochondrial swelling and vacuolization seemed to be typical for Cd treatment, suggesting an impediment of the oxidative metabolism^86^. It also correlates with the increasing level of necrotic cells in both gonads.

Autophagy being responsible for the degradation of organelles and cytoplasmic components, rather than being a type of cell death, can also protect cells against their death^87–90^. However, apoptosis and autophagy can coexist; thus, the distinct cross-talk between these two processes has been described^30,31,55,77,91–93^. The increase in the intensity of autophagy in the cells of both gonads after short-term treatment of the animals with cadmium proves that protective processes are in progress. During autophagy different organelles, e.g., mitochondria, the cisterns of the endoplasmic reticulum, spherites, etc., are neutralized inside autophagosomes due to intracellular digestion, leading to cell survival^89,90^. Autophagy in the 45Cd group turns out to be a less active process compared to the short-term treatment of the animals with cadmium. This correlates with an increase in the intensity of apoptosis in the testis and ovary. As in the case of organs such as the midgut and salivary glands^30,31^, there is a clear cross-talk between these two processes. Our research confirms the observation that autophagy is a common process that enables the functioning of both types of cells (the somatic and germ cells) in testes and ovaries^64,76,77,94,95^. The concentration of ATP was significantly reduced in both gonads after short- and long-term exposure to Cd. We obtained similar results for midgut epithelium^30^. It confirms the possibility of intensification of degenerative processes in cells of both gonads. The highest level of the ADP/ATP index was detected in testes of individuals exposed to cadmium for 12 days. It could relate to the fact that after short-term exposure to cadmium significant differences in the percentage of early apoptotic cells and necrotic cells appeared. The significantly low ATP levels favor necrosis^96,97^.

Our research raised further questions: (1) Does cadmium ingested with food cause similar changes to those observed in soil? (2) Is the number of eggs laid by cadmium-treated females the same as in the control group? (3) Is the success at hatching eggs the same in cadmium-treated and untreated animals? (4) Do the offspring of females treated with cadmium survive puberty in a similar ratio as the offspring of females not treated with this heavy metal? Answering these questions will allow a better understanding of the effects of cadmium on the reproductive abilities of *L. forficatus* and other soil animals. Therefore, more research is needed to explore this problem.

## Conclusions

Our studies showed that (1) cadmium causes damage to the gonad (ovary and testis) structure and affects gametogenesis; (2) male germ-line cells are more sensitive to cadmium than female germ-line cells what is probably related to the accumulation of spherites in ovaries; (3) somatic cells of both gonads play a protective role against heavy metals; (4) distinct cross-talk between autophagy and apoptosis exists in both gonads.

## References

[1] Sieńczuk W (1999). Toksykologia.

[2] Kabata-Pendias A, Pendias H (1999). Biochemia pierwiastków śladowych.

[3] Sharma H, Rawal N, Mathew BB (2015). The characteristics, toxicity and effects of cadmium. Int. J. Nanosci. Nanotechnol..

[4] Duarte A, Cachada A, Rocha-Santos T (2017). Soil pollution: From Monitoring to Remediation.

[5] Zhang H, Reynolds M (2019). Cadmium exposure in living organisms: A short review. Sci. Total Environ..

[6] Lane TW, Saito MA, George GN, Pickering IJ, Prince RC, Morel FMM (2005). A cadmium enzyme from a marine diatom. Nature.

[7] Jӓrup L (2003). Hazards of heavy metal contamination. Br. Med. Bull..

[8] Massányi P, Massányi M, Madeddu R, Stawarz R, Lukáč N (2020). Effects of cadmium, lead, and mercury on the structure and function of reproductive organs. Toxics.

[9] Roy S, Hasanuzzaman M, Prasad MNV, Fujita M (2019). Cadmium accumulation in crops and the increasing risk of dietary cadmium exposure: An overview. Cadmium Tolerance in Plants: Agronomic, Molecular, Signaling, and Omic Approaches.

[10] Templeton DM, Liu Y (2010). Multiple roles of cadmium in cell death and survival. Chem. Biol. Interact..

[11] Stojsavljević A, Rovčanin B, Krstić Đ, Borković-Mitić S, Paunović I, Kodranov I, Gavrović-Jankulović M, Manojlović D (2019). Evaluation of trace metals in thyroid tissues: Comparative analysis with benign and malignant thyroid diseases. Ecotoxicol. Environ. Saf..

[12] Lewis JGE (1981). The Biology of Centipedes.

[13] Hopkin SP (1989). Ecophysiology of Metals in Terrestrial Invertebrates.

[14] Hopkin SP, Read HJ (1992). The Biology of Millipedes.

[15] Lipovšek S, Letofsy-Papst I, Hofer F, Pabst MA (2002). Seasonal- and age-dependent changes of the structure and chemical composition of the spherites in the midgut gland of the harvestmen *Gyas annulatus* (Opiliones). Micron.

[16] Chajec Ł, Rost-Roszkowska MM, Vilimova J, Sosinka A (2012). Ultrastructure and regeneration of midgut epithelial cells in *Lithobius forficatus* (Chilopoda, Lithobiidae). Invertebr. Biol..

[17] Hopkin SP, Watson K, Martin MH, Mould ML (1985). The assimilation of heavy metals by *Lithobius variegatus* and *Glomeris marginata* (Chilopoda; Diplopoda). Bijdr. Dierkd..

[18] Adiyodi KG, Adiyodi RG (1983). Reproductive Biology of Invertebrates. Volume I. Oogenesis, Oviposition, and Oosorption.

[19] Adiyodi KG, Adiyodi RG (1983). Reproductive Biology of Invertebrates. Volume II. Spermatogenesis and Sperm Function.

[20] Sareen ML, Adiyodi KG, Adiyodi KG, Adiyodi RG (1983). Arthropoda – Myriapoda. Reproductive Biology of Invertebrates. Volume I. Oogenesis, Oviposition, and Oosorption.

[21] Minelli A, Minelli A (2011). Chilopoda – Reproduction. Treatise on Zoology – Anatomy, Taxonomy, Biology. The Myriapoda. Vol. 1. Chilopoda.

[22] Parolini M (2020). Toxicity of the non-steroidal anti-inflammatory drugs (NSAIDs) acetylsalicylic acid, paracetamol, diclofenac, ibuprofen and naproxen towards freshwater invertebrates: A review. Sci. Total Environ..

[23] Nath V (1924). Oogenesis of *Lithobius forficatus*. Biol. Rev..

[24] Nath V (1925). Spermathogenesis of *Lithobius forficatus*. Biol. Rev..

[25] Descamps M (1971). Etude ultrastructurale des spermatogonies et de la croissance spermatocytaire chez *Lithobius forficatus* L. (Myriapode Chilopode). Z. Zellforsch..

[26] Descamps M (1971). Le cycle spermatogenétique chez *Lithobius forficatus* L. (Myriapode, Chilopode). I. Evolution et etude quantitative des populations cellulaires du tes ticle au cours du développement post-embryonnaire. Arch. Zool. Exp. Gen..

[27] Herbaut C (1972). Etude cytochimique et ultrastructurale de l'ovogenése chez *Lithobius forficatus* L. (Myriapode Chilopode). Evolution des constituants cellulaires. Wilhelm Roux' Arch..

[28] Descamps M, Fabre MC, Grelle C, Gerard S (1996). Cadmium and lead kinetics during experimental contamination of the centipede *Lithobius forficatus* L. Arch. Environ. Contam. Toxicol..

[29] Vandenbulcke F, Grelle C, Fabre M-C, Descamps M (1998). Implication of the midgut of the centipede *Lithobius forficatus* in the heavy metal detoxification process. Ecotoxicol. Environ. Saf..

[30] Rost-Roszkowska M (2020). Influence of soil contaminated with cadmium on cell death in the digestive epithelium of soil centipede *Lithobius forficatus* (Myriapoda, Chilopoda). Eur. Zool. J..

[31] Rost-Roszkowska M, Poprawa I, Chajec Ł, Chachulska-Żymełka A, Leśniewska M, Student S (2020). Effects of short- and long-term exposure to cadmium on salivary glands and fat body of soil centipede *Lithobius forficatus* (Myriapoda, Chilopoda): Histology and ultrastructure. Micron.

[32] Rost-Roszkowska M (2021). Effects of cadmium on mitochondrial structure and function in different organs: Studies on the soil centipede *Lithobius forficatus* (Myriapoda, Chilopoda). Eur. Zool. J..

[33] Włodarczyk A, Student S, Rost-Roszkowska M (2019). Autophagy and apoptosis in starved and refed *Neocaridina davidi* (Crustacea, Malacostraca) midgut. Can. J. Zool..

[34] Bradford MM (1976). Rapid and sensitive method for the quantitation of microgram quantities of protein utilizing the principle of protein-dye binding. Anal. Biochem..

[35] Wieser W (1967). Conquering terra firma: The copper problem from the isopod’s point of view. Helgolander Wiss. Meeresunters..

[36] Gräff S, Berkus M, Alberti G, Köhler HR (1997). Metal accumulation strategies in saprophagous and phytophagous soil invertebrates: A quantitative comparison. Biometals.

[37] Siekierska E, Urbańska-Jasik D (1998). The effect of cadmium and selenium ions on the ovary structure in leech *Herpobdella octooculata* (L.). Folia Morphol..

[38] Siekierska E, Urbańska-Jasik D (2002). Cadmium effect on the ovarian structure in earthworm *Dendrobaena veneta* (Rosa). Environ. Pollut..

[39] Osman W, El-Samad LM, Mokhamer EL-H, El-Touhamy A, Shonouda M (2015). Ecological, morphological, and histological studies on *Blaps polycresta* (Coleoptera: Tenebrionidae) as biomonitors of cadmium soil pollution. Environ. Sci. Pollut. Res. Int..

[40] Siekierska, E. & Brzozowa, M. Cadmium effect on the seminal vesicles structure and spermatogenesis in the earthworm *Dendrobaena veneta* (Rosa). In *8th International Symposium on Earthworm Ecology*. *Book of abstracts,* 231 (2006).

[41] Siekierska E, Brzozowa M (2008). Changes in primary and secondary spermatocytes in seminal vesicles in the earthworm *Dendrobaena veneta* (Rosa) after 10 days of cadmium exposure. Acta Biol. Cracov. Bot..

[42] Brzozowa, M. Wpływ kadmu na przebieg spermiogenezy u dżdżownicy *Dendrobaena veneta* (Rosa). PhD Thesis, University of Silesia in Katowice Poland (2009).

[43] Papathanassiou E (1986). Cadmium accumulation and ultrastructural alterations in oogenesis of the prawn *Palaemon serratus* (Pennant). Bull. Environ. Contam. Toxicol..

[44] Au DWT, Chiang MWL, Wu R (2000). Effect of cadmium and phenol on mortality and ultrastructure of sea urchin and mussel spermatozoa. Arcg. Environ. Contam. Toxicol..

[45] Au DWT, Lee CY, Chan KL, Wu R (2001). Reproductive impairment of sea urchins upon chronic exposure to cadmium. Part I: Effects on gamete quality. Environ. Pollut..

[46] Au DWT, Reunov AA, Wu R (2001). Reproductive impairment of sea urchins upon chronic exposure to cadmium. Part II: Effects on sperm development. Environ. Pollut..

[47] Eckelbarger KJ (1994). Diversity of metazoan ovaries and vitellogenic mechanisms – implications for life history theory. Proc. Biol. Soc. Wash..

[48] Suzuki KT, Yamamura M, Mori T (1980). Cadmium-binding proteins induced in earthworm. Arch. Environ. Contam. Toxicol..

[49] Maroni G, Wise J, Young JE, Otto E (1987). Metallothionein gene duplications and metal tolerance in natural populations of *Drosophila melanogaster*. Genetics.

[50] Luo M, Finet C, Cong H, Wei H, Chung H (2020). The evolution of insect metallothioneins. Proc. R. Soc. B.

[51] Turbeck BO (1974). A study of the concentrically laminated concretions, ‘spherites’, in the regenerative cells of the midgut of Lepidopterous larvae. Tissue Cell..

[52] Cruz-Landim C (2000). Localization of calcium and acid phosphatase in the Malpighian tubules of nurse workers of *Melipona quadrifasciata anthidioides* Lep. (Hymenoptera, Apidae, Meliponini). Biosci. J..

[53] Lipovšek S, Letofsky-Papst I, Hofer F, Pabst MA, Devetak D (2012). Application of analytical electron microscopic methods to investigate the function of spherites in the midgut of the larval antlion *Euroleon nostras* (Neuroptera: Myrmeleontidae). Microsc. Res. Tech..

[54] Pinheiro DO, Conte H, Gregório EA (2008). Spherites in the midgut epithelial cells of the sugarcane borer parasitized by *Cotesia flavipes*. Biocell.

[55] Rost-Roszkowska MM, Kszuk-Jendrysik M, Marchewka A, Poprawa I (2018). Fine structure of the midgut epithelium in the millipede *Telodeinopus aoutii* (Myriapoda, Diplopoda) with special emphasis on epithelial regeneration. Protoplasma.

[56] Lipovšek S, Novak T, Dariš B, Hofer F, Leitinger G, Letofsky-Papst I (2022). Ultrastructure of spherites in the midgut diverticula and Malpighian tubules of the harvestman *Amilenus aurantiacus* during the winter diapause. Histochem. Cell Biol..

[57] Kramarz P (1999). Dynamics of accumulation and decontamination of cadmium and zinc in carnivorous invertebrates. 2. The centipede *Lithobius mutabilis* Koch. Bull. Environ. Contam. Toxicol..

[58] Rost-Roszkowska MM, Vilimová J, Tajovský K, Šustr V, Ostróżka A, Kaszuba F (2021). Structure of the midgut epithelium in four diplopod species: Histology, histochemistry and ultrastructure. Arthropod Syst. Phylogeny.

[59] Köhler H-R (2002). Localization of metals in cells of saprophagous soil arthropods (Isopoda, Diplopoda, Collembola). Microsc. Res. Tech..

[60] Cervera A, Maymó AC, Martínez-Pardo R, Garcerá MD (2005). Vitellogenesis inhibition in *Oncopeltus fasciatus* females (Heteroptera: Lygaeidae) exposed to cadmium. J. Insect Physiol..

[61] Cervera A, Maymó AC, Martínez-Pardo R, Garcerá MD (2006). Vitellogenin polypeptide levels in one susceptible and one cadmium-resistant strain of *Oncopeltus fasciatus* (Heteroptera: Lygaeidae), and its role in cadmium resistance. J. Insect Physiol..

[62] Sehgal A, Osgood C, Zimmering S (1990). Aneuploid in Drosophila. III: Aneuploidogens inhibit in vitro assembly of taxol-purified Drosophila microtubules. Environ. Mol. Mutagen..

[63] Li W, Zhao Y, Cou IN (1993). Alterations in cytoskeletal protein sulfhydryls and cellular glutathione in cultured cells exposed to cadmium and nickel ions. Toxicology.

[64] dos Santos DC, Gregorio EA, Moreli Silva de Moraes RL (2007). Programmed cell death during early oogenesis in the *Diatraea saccharalis* germarium. Acta Microsc..

[65] Hoeppner DJ, Hengartner MO, Schnabel R (2001). Engulfment genes cooperate with ced-3 to promote cell death in *Caenorhabditis elegans*. Nature.

[66] Hikim APS (2003). Key apoptotic pathways for heat-induced programmed germ cell death in the testis. Endocrinology.

[67] Russell LD, Chiarini-Garcia H, Korsmeyer SJ, Knudson CM (2002). Bax-dependent spermatogonia apoptosis is required for testicular development and spermatogenesis. Biol. Reprod..

[68] Shaha C, Tripathi R, Mishra DP (2010). Male germ cell apoptosis: Regulation and biology. Philos. Trans. R. Soc. Lond. B Biol. Sci..

[69] Devine PJ, Payne CM, McCuskey MK, Hoyer PB (2000). Ultrastructural evaluation of oocytes during atresia in rat ovarian follicles. Biol. Reprod..

[70] Hussein MR (2005). Apoptosis in the ovary: Molecular mechanisms. Hum. Reprod. Update.

[71] Miller MA, Technau U, Smith KM, Steele RE (2000). Oocyte development in *Hydra* involves selection from competent precursor cells. Dev. Biol..

[72] Matova N, Cooley L (2001). Comparative aspects of animal oogenesis. Dev. Biol..

[73] Technau U, Miller MA, Bridge D, Steele RE (2003). Arrested apoptosis of nurse cells during *Hydra* oogenesis and embryogenesis. Dev. Biol..

[74] Mpakou VE, Nezis IP, Stravopodis DJ, Margaritis LH, Papassideri IS (2006). Programmed cell death of the ovarian nurse cells during oogenesis of the silkmoth *Bombyx mori*. Dev. Growth Differ..

[75] Mpakou VE (2008). Different modes of programmed cel death during oogenesis of the silkmoth *Bombyx mori*. Autophagy.

[76] Mpakou VE, Velentzas AD, Velentzas PD, Margaritis LH, Stravopodis DJ, Papassideri IS (2011). Programmed cell death of the ovarian nurse cells during oogenesis of the ladybird beetle *Adalia bipunctata* (Coleoptera: Coccinellidae). Dev. Growth Differ..

[77] Poprawa I, Hyra M, Kszuk-Jendrysik M, Rost-Roszkowska MM (2015). Ultrastructural changes and programmed cell death of trophocytes in the gonad of *Isohypsibius granulifer granulifer* Thulin, 1928 (Tardigrada, Eutardigrada, Isohypsibiidae). Micron.

[78] Janelt K, Jezierska M, Poprawa I (2019). The female reproductive system and oogenesis in *Thulinius ruffoi* (Tardigrada, Eutardigrada, Isohypsibiidae). Arthropod. Struct. Dev..

[79] Mooyottu S, Anees C, Cherian S (2011). Ovarian stem cells and neo-oogenesis: A breakthrough in reproductive biology research. Vet. World.

[80] Tiwari M (2015). Apoptosis in mammalian oocytes: A review. Apoptosis.

[81] Xiu Y-R, Yang W-X (2018). Roles of three Es-Caspases during spermatogenesis and cadmium-induced apoptosis in *Eriocheir sinensis*. Aging.

[82] Redza-Dutordoir M, Averill-Bates DA (2016). Activation of apoptosis signalling pathways by reactive oxygen species. Biochim. Biophys. Acta.

[83] Sonakowska L, Włodarczyk A, Wilczek G, Wilczek P, Student S, Rost-Roszkowska MM (2016). Cell death in the epithelia of the intestine and hepatopancreas in *Neocaridina heteropoda* (Crustacea, Malacostraca). PLoS ONE.

[84] Włodarczyk A (2017). The effect of starvation and re-feeding on mitochondrial potential in the midgut of *Neocaridina davidi* (Crustacea, Malacostraca). PLoS ONE.

[85] Zorova LD (2018). Mitochondrial membrane potential. Anal. Biochem..

[86] Ossola JO, Tomaro ML (1995). Heme oxygenase induction by cadmium chloride: Evidence for oxidative stress involvement. Toxicology.

[87] Levine B, Klionsky DJ (2004). Development by self-digestion: Molecular mechanisms and biological functions of autophagy. Dev. Cell..

[88] Kourtis N, Tavernarakis N (2009). Autophagy and cell death in model organisms. Cell Death Differ..

[89] Kliosnky D (2016). Guidelines for the use and interpretation of assays for monitoring autophagy (3rd edition). Autophagy.

[90] Kliosnky D (2021). Guidelines for the use and interpretation of assays for monitoring autophagy (4th edition). Autophagy.

[91] Velentzas AD, Nezis IP, Stravopodis DJ, Papassideri IS, Margaritis LH (2007). Apoptosis and autophagy function cooperatively for the efficacious execution of programmed nurse cell death during *Drosophila virilis* oogenesis. Autophagy.

[92] Lipovšek S (2018). Changes in the midgut cells in the European cave spider, *Meta menardi*, during starvation in spring and autumn. Histochem. Cell Biol..

[93] Rost-Roszkowska MM (2019). Autophagy and apoptosis in the midgut epithelium of millipedes. Microsc. Microanal..

[94] Nezis IP (2010). Autophagy as a trigger for cell death: Autophagic degradation of inhibitor of apoptosis dBruce controls DNA fragmentation during late oogenesis in *Drosophila*. Autophagy.

[95] Rost-Roszkowska MM, Janelt K, Poprawa I (2018). The role of autophagy in the midgut epithelium of Parachela (Tardigrada). Zoomorphology.

[96] Leist M, Single B, Castoldi AF, Kühnle S, Nicotera P (1997). Intracellular adenosine triphosphate (ATP) concentration: A switch in the decision between apoptosis and necrosis. J. Exp. Med..

[97] Nikoletopoulou V, Markaki M, Palikaras K, Tavernarakis N (2013). Crosstalk between apoptosis, necrosis and autophagy. Biochim. Biophys. Acta..

